# RBM10, a New Regulator of p53

**DOI:** 10.3390/cells9092107

**Published:** 2020-09-16

**Authors:** Ji Hoon Jung, Hyemin Lee, Shelya X Zeng, Hua Lu

**Affiliations:** 1 College of Korean Medicine, Kyung Hee University, Seoul 02447, Korea; 2 Department of Biochemistry and Molecular Biology, Tulane University School of Medicine, New Orleans, LA 70112, USA; hlee17@tulane.edu (H.L.); szeng@tulane.edu (S.X.Z.); 3 Tulane Cancer Center, Tulane University School of Medicine, New Orleans, LA 70112, USA

**Keywords:** RBM10, p53, apoptosis, MDM2, cancer

## Abstract

The tumor suppressor p53 acts as a transcription factor that regulates the expression of a number of genes responsible for DNA repair, cell cycle arrest, metabolism, cell migration, angiogenesis, ferroptosis, senescence, and apoptosis. It is the most commonly silenced or mutated gene in cancer, as approximately 50% of all types of human cancers harbor TP53 mutations. Activation of p53 is detrimental to normal cells, thus it is tightly regulated via multiple mechanisms. One of the recently identified regulators of p53 is RNA-binding motif protein 10 (RBM10). RBM10 is an RNA-binding protein frequently deleted or mutated in cancer cells. Its loss of function results in various deformities, such as cleft palate and malformation of the heart, and diseases such as lung adenocarcinoma. In addition, RBM10 mutations are frequently observed in lung adenocarcinomas, colorectal carcinomas, and pancreatic ductal adenocarcinomas. RBM10 plays a regulatory role in alternative splicing. Several recent studies not only linked this splicing regulation of RBM10 to cancer development, but also bridged RBM10′s anticancer function to the p53 pathway. This review will focus on the current progress in our understanding of RBM10 regulation of p53, and its role in p53-dependent cancer prevention.

## 1. Introduction

p53 is one of the best-known tumor suppressors. It is the most popularly researched protein in the field of biomedical sciences, as a continuingly increasing number of research and review articles have been published about p53, in all disciplines of biomedical research, biochemistry, genetics, cellular and molecular biology, pharmacology, clinical research, immunology, and cancer research, since its discovery in 1979 [1,2]. p53 is able to protect normal cells from tumorigenesis in response to a wide range of stimuli, such as ribosomal dysfunction, oxidative stress, DNA damage, lack of nutrients, hypoxia, and oncogenic activation [3,4,5,6]. Once activated, p53 induces apoptosis, ferroptosis, senescence, and cell cycle arrest; and inhibits tumor angiogenesis, cancer cell migration, and metastasis [6,7]. Apoptosis is achieved in part through transcriptional activation of apoptotic genes, which is dependent on offending stressors [8]. Mitochondrial-dependent apoptosis ensures following upregulation of mitochondrial proteins, such as Bax, PUMA, and NOXA [9,10,11]. Furthermore, the autophagy regulator DRAM1 may play a role in p53-dependent apoptosis [12]. Independent of its transcriptional activity, p53 can also function outside of the nucleus, to directly inhibit the activity of anti-apoptotic proteins Bcl-2 and Bcl-x_L_ in the mitochondria [13]. p21, also called WAF1, is an important component of the p53 pathway. Once DNA is damaged, the transcription of p21 is stimulated by p53, resulting in the G1 cell cycle arrest [14,15]. p21 also attenuates EMT (epithelial-mesenchymal transition), a biological process that allows epithelial cells, with loss of cell polarity and cell–cell adhesion via various molecular and biochemical mechanisms, to migrate to distant tissues as mesenchymal cells, by inhibiting the functions of RAS and c-Myc [16]. The importance of p53′s anti-cancer functions in preventing tumor progression is exceedingly highlighted by the high frequency of its functional aberration in human cancers that either harbor mutations in its gene (over 50%) or highly express its oncogenic inhibitor molecules, such as MDM2 or MDMX. Often, mutated p53s, such as hot spot mutants, gain a new oncogenic function [17,18,19,20]. Hence, this p53 pathway is subjected to multi-level regulations.

One of the recently identified p53 regulators is RNA-binding motif protein 10 (RBM10; also called S1-1). RBM10 is a member of the RNA binding motif gene family and was first discovered as DXS8237E [21,22] and located on p11.3 of the X chromosome. It has been shown to be involved in pre-mRNA (messenger RNAs) splicing and posttranscriptional regulation [23]. RBM10, a protein with 930 amino acids, contains two RNA recognition motifs (RRM), two zinc fingers, and one G patch motif [24]. RBM10 partially exists in nuclear speckles, and in perichromatin fibrils as the S1-1 granules [25,26], which is correlated with its activity in regulation of gene transcription, mRNA alternative splicing, and stabilization of various genes, including apoptosis related genes [27,28,29,30]. RBM10 has been shown to be a key player in the TARP (Talipes equinovarus, Atrial septal defect, Robin sequence, and Persistent left superior vena cava) syndrome, as its mutations are highly associated with this syndrome [23,31]. This syndrome is a rare condition that affects males (due to one copy X chromosome) and causes several birth defects, such as club foot deformity, congenital heart defects, a smaller jaw, and a retracted or displaced tongue, as reflected in its definition, or name, above. Biochemically, RBM10 has been shown to act as an alternative splicing factor of several pre-mRNAs, such as VEGF [32], Dlg4 [33], FAS, Bcl-x [25], SMN2 [34], and NUMB [35]. RBM10 also possesses splicing-independent functions. For instance, it was shown to mediate an anti-hypertrophy action by associating with Star-PAP (TUT1). This association led to temporary stabilization of angiotensin II type 1 (AT1) receptor mRNA, but reduction of its transcription, consequently inhibiting proliferation, and increasing apoptosis, of vascular smooth muscle cells (VSMC) [36]. RBM10 can also regulate filamin A (FLNa)-binding RhoGTPase-activating protein (FilGAP) activity after translocating from the nucleus into the cellular peripheries by Fyn in response to Src tyrosine kinase signaling [37]. RBM10 has been shown to be involved in potential regulation of transcription of a number of genes [38], though the mechanisms and the importance of these regulations remain to be investigated. This review will focus on recent studies on the regulation of the p53 pathway by RBM10.

## 2. The MDM2-MDMX-p53 Loop

Due to the cytotoxicity of highly active p53, this tumor suppressor needs to be tightly controlled in normal cells. Indeed, unstressed and non-transformed normal cells contain low levels of less potent wild type p53, due to its short half-life [39,40]. This is because a p53 responsive gene encodes a Ring-Finger E3 ubiquitin ligase called MDM2 [41]. Biochemically, MDM2 utilizes at least two mechanisms to inactivate p53. On the one hand, MDM2 binds to the N-terminal domain of p53 and mediates p53 ubiquitination and consequent degradation by the 26 S proteasome [42,43,44]. MDM2 binding to the NH2 terminal transactivation domain of p53 blocks its transcriptional activity via its own N-terminal domain with a hydrophobic pocket. Accordingly, the mutations in the MDM2 binding site of p53 are resistant to proteasomal degradation mediated by MDM2. Similarly, MDM2 mutations that lack p53 binding fail to degrade p53 [45]. MDMX (also called MDM4), an analog of MDM2 [46], further enhances the MDM2 activity to polyubiquitinate p53 by forming a complex with MDM2, through their respective RING domains [44,47,48,49]. Intriguingly, MDMX itself is a substrate of the MDM2 E3 ligase, thus acting as a competitive substrate for polyubiquitination against p53 [47,50,51]. On the other hand, MDM2 and MDMX can also cooperatively and independently suppress p53′s transcriptional activity by binding to the N-terminal transcriptional domain of p53 via their own N-termini [52]. The mutual dependence of MDM2 and MDMX for their p53-inactivating functions, as well as their essential roles in controlling p53 level and activity in vivo, has been demonstrated in several mouse genetic studies [53,54,55,56,57,58]. Therefore, because both MDM2 and MDMX are p53 responsive transcriptional targets, they play essential roles in controlling p53 level and activity in a negative feedback fashion during embryogenesis and organogenesis. In order to activate p53, it is necessary to overcome this negative feedback regulation. Indeed, it has been shown that diverse stress stimuli, including activation of oncogenes, DNA damage by agents or nutrient deprivation, lead to the drastic increase of p53 levels and activity by inhibiting MDM2 or MDMX activity. In general, this is executed through either increasing expression, and posttranslational modifications, of MDM2 or MDMX, or posttranslational modifications of p53 itself, such as phosphorylation, acetylation, and so on. Consequently, active p53 exerts its cellular functions, as highlighted above, primarily by regulating the expression of its target genes [59]. Thus, this MDM2-MDMX-p53 loop is subjected to a tight regulation through multiple cellular and molecular pathways, and mechanisms, as further detailed below.

## 3. Regulation of the p53-MDM2-MDMX Loop

Over the past decades, a number of proteins have been identified to regulate this MDM2-MDMX-p53 feedback loop upon stresses. For example, in response to DNA damage caused by chemotherapeutic agents, p53 is post-translationally modified, including phosphorylation by various kinases, such as ATM, ATR, DNA-PK, Chk1, Chk, CDK7/Cyc H, and CKII-FACT [60]. DNA damage signals have also been shown to activate p53 by inducing its acetylation, which is mediated by several histone acetyl-transferases (HATs), including p300, CBP, PCAF, TIP60 and so on [61,62,63,64,65,66,67]. Acetylation of p53 not only enhances its transcriptional activity, but also blocks its ubiquitination mediated by MDM2 as both p300/CBP and MDM2 often target a similar set of lysine residues of p53, and thus these two types of posttranslational modifications (PTM) are mutually exclusive [65,68,69,70]. Also, in response to oncogenic challenges, p53 becomes stabilized through the antagonism of the p53–Mdm2 interaction by p14^ARF^ (p19^ARF^ for mouse), whose transcription can be induced via oncogenic signals, such as activation of E2F1 and c-Myc [71,72,73].

In addition, PTMs of MDM2 and MDMX have been shown to play roles in regulation of this MDM2-MDMX-p53 loop. On the one hand, in response to HER-2/neu-PI3K signaling, ATK became active to phosphorylate MDM2 at several serines and render the phosphorylated MDM2 imported to the nucleus, consequently inactivating p53 [74,75,76,77,78]. On the other hand, in response to DNA damage signals, ATM phosphorylates MDM2 at serine 395, suppressing MDM2 activity toward p53 and consequently activating the latter [79]. Thus, this MDM2-p53 pathway is highly regulated via a variety of signaling pathways, as further discussed below and reviewed by others recently [80,81,82,83]. Furthermore, ATM/ATR-Chk2/Chk1 kinase cascades can lead to MDMX phosphorylation at S342, S367, and S402 in response to DNA damage [84,85,86,87]. This phosphorylation, particularly at S342 and S367, results in the binding of MDMX to 14-3-3, and this binding leads to inactivation of MDMX, consequently activating p53 [84,85,86,87]. This pathway was elegantly validated by a genetic study showing that mutations at S341 (equivalent to human S342), S367, and S402 of mouse MDMX (MDMX-3SA) impair the p53 response to DNA damage signals caused by gamma irradiation in mice [88].

### 3.1. Ribosomal Proteins-p53-MDM2 Pathway

Ribosome stress is caused by any stressing signals and molecular or genetic alterations that disrupt the ribosomal biogenesis in the nucleolus of a cell. This de novo biogenesis is generally comprised of three steps in the nucleolus: (1) rRNA synthesis by RNA polymerase I, (2) rRNA processing by RNA-protein complexes via several steps, with yet incompletely understood mechanisms, and (3) 40S and 60S ribosomes assembly. Thus, constraining any of these steps would lead to ribosomal stress. These stressors include treatment of cells with actinomycin D (Act D) [89], 5-fluorouracil (5-FU) [90], or mycophenolic acid (MPA) [91], serum starvation [92], impairment of 40S or 60S ribosomal biogenesis by knockdown of either ribosomal protein, a nucleolar protein important for rRNA processing [93]. Upon ribosomal stress, several RPs, such as RPL5 [6,94], RPL11 [92,95], RPL22 [96], RPL23 [97], RPL26 [98], RPS14 [99], or RPS27a [100] are free from the ribosomes in the nucleolus to the nucleoplasm where they find MDM2 to bind, and consequently inhibit MDM2 activity toward p53. These ribosomal proteins, such as RPL5, RPL11, RPL23, RPL26, RPS7, or RPS14 generally bind to the central domain of MDM2 [101], in a way similar to ARF does [73], and inhibit its Ring finger E3 ubiquitin ligase activity to p53. For instance, it has been shown that RPL11 and RPL5 can form a heterodimer via 5S rRNA and bind to the Zinc finger domain within the central region of MDM2 via several hydrophilic residues within this domain [102,103]. This binding renders the conformational change of the C-terminal domain of MDM2 and makes it less active in terms of ubiquitinating p53 [7,8,104]. However, it still remains to be investigated how exactly the RPL5-5S rRNA-RPL11 complex as well other ribosomal proteins bind to MDM2 and inactivate its function toward p53, in response to ribosomal stress. This ribosomal stress pathway has been validated genetically [6,105,106,107,108,109]. Recently, our lab has identified several nucleolar proteins that can regulate this ribosomal stress-RPL11/RPL5-MDM2-p53 pathway. For example, SPIN1, a new member of the SPIN/SSTY family, was identified as a new RPL5 inhibitory regulator. SPIN1 is important for the MDM2-p53 pathway via being a binding partner of human RPL5. SPIN1 prevents L5 interacting with MDM2, and thereby L5-mediated inhibition of MDM2 ubiquitin ligase activity toward p53 [110]. The nucleolar protein nucleostemin (NS) is essential for cell proliferation and early embryogenesis. Additionally, NS regulates p53 activity through the inhibition of MDM2. These effects require the ribosomal proteins L5 and L11, since the depletion of NS enhanced their interactions with MDM2 and the knockdown of L5 or L11 abrogated the NS depletion-induced p53 activation [111,112].

### 3.2. AMPK-MDMX-p53 Pathway in Cancer Cells

The cancer cells undergo alternations of metabolism of glucose and lipid, and oxidative responses. These processes are also involved in AMPK and p53 [113,114]. AMPK is activated by LKB1 and regulates autophagy, metabolism, cell proliferation, and apoptosis by phosphorylating its target proteins. Initially, AMPK was found to be activated in response to glucose starvation leading to p53 activation, via yet unidentified mechanisms [114]. Later on, AMPK was shown to phosphorylate MDMX at Ser342 (mouse Ser341), and this phosphorylation led to the association of MDMX with 14-3-3, subsequently inactivating MDMX activity toward p53, with an ultimate activation of the latter [113]. Genetic studies demonstrate that mutation of MDM2 (C305F), impairing the ribosomal stress signaling [4] and mutation of MDMX (3SA), impairing the DNA damage signaling [115], can lead to defects of lipid and glucose metabolisms in cancer cells [1,116]. These lines of information would offer molecular insights into how glucose metabolism is controlled by the AMPK-MDM2/MDMX-p53 pathway in cancer cells, and potential molecule targets in the pathway, for future development of anti-cancer drugs.

**Figure 1:**
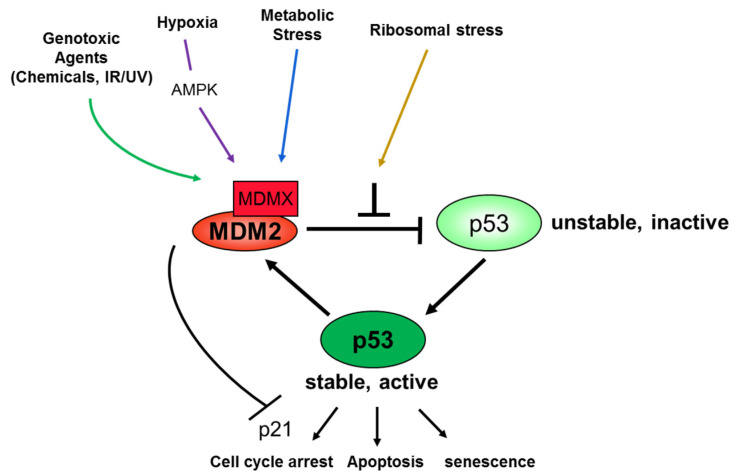
Schematic for regulation of MDM2-MDMX-p53 loop by stress signals. Arrow = activation; Bar = inhibition.

Therefore, the MDM2-MDMX-p53 pathway is subjected to multi-layers of regulations in response to various stress signals (Figure 1), involving a plethora of distinct protein molecules. One of the recently identified regulators of this pathway is RBM10.

## 4. Tumor Suppressive Functions of RBM10 in Cancer Cells

**Figure 2:**
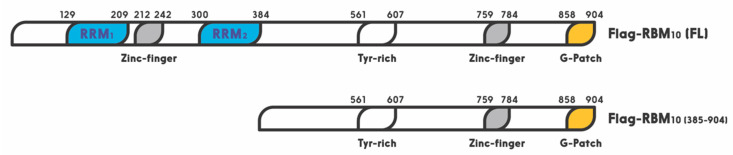
Schematic for functional domains of RNA-binding motif protein 10 (RBM10). Numbers indicate the positions of amino acids.

RBM10 is a member of the large RNA binding protein family that regulates all aspects of RNA metabolism [38]. Especially, RBM10 has been shown to act as a regulator of alternative splicing [34,117]. A recent study showed that miR-335 modulates Numb alternative splicing via targeting RBM10 in endometrial cancer [118]. In this family, RBM10 shares a highly similar RNA-binding motif and a 30–50% amino acid sequence identity, with two other members, RBM5 and RBM6 [35]. The RBM10 gene was first cloned from human bone marrow in 1995, and later from rat liver in 1996. Its 3.5 kb OFR stems from 24 exons. This X chromosome-linked RBM10 gene encodes a 930 amino acid protein with two RNA recognition motifs (RRM1 and RRM2), two zinc fingers, and one G patch motif [24] (Figure 2). It is widely expressed in a number of types of cells and regulates gene transcription, mRNA alternative splicing, and stabilization of various genes, including the apoptosis related gene [27,28,29]. Its expression and mutations are highly associated with TARP syndrome that causes high lethality to children [23]. It is also associated with spinal muscular atrophy (SMA) [34].

Interestingly, all of the three RBM proteins have been shown to play a potential role in preventing tumorigenesis. For example, RBM5 and RBM6 are frequently deleted in lung cancer and heavy smokers. First of all, RBM5 is downregulated in about 75% of lung cancers as well as prostate and breast cancers [35,119]. Interestingly, overexpression of RBM5 was also reported to play dual functions in breast cancer [35]. Specifically, RBM5 has been shown to regulate alternative splicing of apoptosis related genes, Fas receptor and c-FLIP, resulting in control of programmed cell death [25,27,119]. Recent studies showed that RBM5 is regulated by long noncoding RNAs (lncRNAs) or miRNAs in cancer cells. For instance, the long noncoding RNA AFAP1-AS1 accelerated the proliferation and metastasis of prostate cancer cells by inhibiting RBM5 expression [120]. Additionally, another lncRNA, AB073614, promoted the proliferation and inhibited apoptosis of cervical cancer cells by repressing RBM5 [121]. RBM6 was first identified in small cell lung carcinoma. This gene has been shown to be deleted or disrupted in lung cancers [122]. Furthermore, RBM6 was reported to inhibit invasion and induce apoptosis, consequently suppressing the cancer cell growth and progression in laryngocarcinoma [123]. Together, these lines of evidence suggest that both RBM5 and RBM6 might play a tumor suppressive role in different types of cancers.

More recently, RBM10 was reported to inhibit lung cancer cells cell viability and cell cycle progression [124]. This is well in line with the fact that RBM10 mutations are observed in a number of human cancers, such as lung adenocarcinoma [125,126], pancreatic [127,128], colorectal [129], and thyroid [130] cancers. Accordingly, RBM10 is revealed as a cancer-associated gene and listed in the Catalog of Somatic Mutations in Cancer (COSMIC) database [131]. Mechanistically, RBM10 can induce tumor necrosis factor alpha (TNF-α) and apoptosis [132]. Moreover, it has been recently shown to suppress cancer cell proliferation through its function in alternative splicing of oncogenic mRNAs, such as NUMB [118] and VEGFA [32], or by inhibiting RAP1/AKT/CREB signaling [133]. Furthermore, our recent study showed that over expression of RBM10 reduces cancer cell migration and the EMT related gene Vimentin as well [134]. In addition, our latest study linked the anti-cancer role of RBM10 to its regulation of the MDM2-p53 pathway, as further described below. RBM10 can induce apoptosis of cancer cells by activating p53. Altogether, these studies highly suggest that RBM10, similar to RBM5 and RBM6, possesses a tumor suppressive function via different mechanisms.

## 5. RBM10 is an Activator of p53

As mentioned above, one of the mechanisms underlying the tumor suppressive role of RBM10 is through regulation of the p53-MDM2 feedback loop. Prior to RBM10, several members of the RBM family have been shown to play a role in the p53 pathway [135,136]. For instance, RBM38 was first shown to act as a tumor suppressor by blocking the p53-MDM2 loop in hepatocellular carcinoma [136]. Upregulation of RBM38 induced p53 expression, and decreased MDM2 expression, in human liver cancer cells. However, this regulation was specific to wt p53, as upregulation of RBM38 did not affect the expression of mutant p53 [136]. Another member of this family, RBM24, which shares high similarity with RBM38 in the RRM region, was found to regulate p21 mRNA expression in response to p53 activation, as p53 binds and activates the promoter of the *RBM24* gene [135]. By contrast, RBM25, a potential tumor suppressor, was recently found to bind directly to circAMOTL1L, a circular RNA, and induces its biogenesis as a downstream player of p53 in regulation of EMT, as it is a p53 target gene as well, and its disfunction contributes to prostate cancer progression [137].

**Figure 3:**
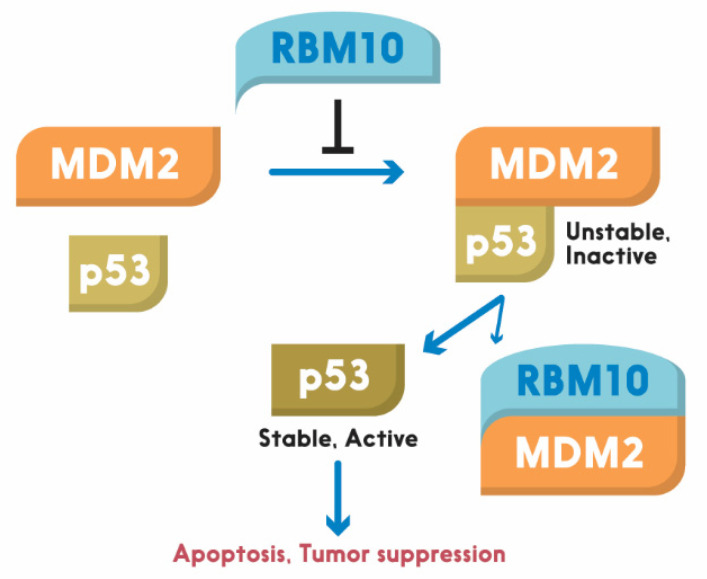
Schematic for RBM10 regulation of the p53-MDM2 loop. Arrow = activation; Bar = inhibition.

Similar to these RBM proteins, RBM10 was recently shown by our group to act as tumor suppression. Its tumor suppression role is well supported by bioinformatics data, as the lower expression of RBM10 is correlated with lower survival rate of colorectal cancer patients [6]. It exerts its anti-cancer function through activation of p53. We showed that overexpression of RBM10 induces p53-dependent apoptosis of various cancer cells. Furthermore, we found that the overexpression of RBM10 can reduce MDM2-mediated ubiquitination and degradation of p53. This inhibition must be executed via the direct interaction between RBM10 and MDM2, as both exogenous, and endogenous, RBM10 and MDM2 can be pulled down via co-immunoprecipitation [134]. This was further confirmed by our domain mapping experiments, showing that the RRM1-Zinc Finger (ZnF) and RRM2 region of RBM10 is crucial for MDM2-binding (Figure 2). This region has also been shown to play an important role in the alternative splicing by RNA motifs in introns or exons of its target RNAs [138]. This domain is also crucial for p53 activation, as deletion of the N-terminal RRM1-ZnF domain leads to partial activation of p53 compared to the full-length RBM10 [134]. Supporting this notion, the ability of mutant RBM10 to bind to MDM2 is reduced as measured by co-IP-IB analysis in cancer cells. Surprisingly, this mutant RBM10 was still able to bind to p53. However, RBM10 does not appear to bind to MDMX. These results suggest that the N-terminal domain of RBM10 is required for MDM2 binding and consequent p53 induction. Therefore, our study unveils RBM10 as another regulator of the MDM2-p53 loop and a tumor suppressor that can inhibit cell proliferation and growth in part by blocking this loop and consequently activating p53 (Figure 3).

## 6. Conclusions and Questions

In conclusion, our recent study, as described above, revealed RBM10 as another p53 activator that can suppress tumor cell growth and proliferation by inducing p53 and its pathway. This anti-cancer function of RBM10 is implemented in part by blocking MDM2-mediated ubiquitination and degradation of p53 [134], though it has also been shown to suppress tumorigenesis by selectively modifying alternative splicing [32]. However, there are still some remaining questions. For instance, it still remains unknown how RBM10 is activated to work on the MDM2-p53 loop, and by what signal. Would disfunction of the alternative splicing machinery lead to p53 activation by utilizing RBM10? In addition, it remains to be investigated if the regulation of the loop by RBM10 has anything to do with its function in alternative splicing [25,117]. Moreover, since RBM10 is an RNA binding protein, would it be possible for this protein to regulate the turnover or stability of p53 RNAs? Finally, does RBM10 play a role in regulation of the splicing processes for p53 or MDM2 RNAs? Solving these enticing puzzles would lead to a better understanding of molecular insights into this pathway, and offer useful information for future development of cancer therapy by targeting the RBM10-MDM2-p53 pathway.
